# Data on formaldehyde sources, formaldehyde concentrations and air exchange rates in European housings

**DOI:** 10.1016/j.dib.2018.11.096

**Published:** 2018-11-24

**Authors:** Tunga Salthammer

**Affiliations:** Fraunhofer WKI, Department of Material Analysis and Indoor Chemistry, Bienroder Weg 54 E, 38108 Braunschweig, Germany

## Abstract

Formaldehyde has been discussed as a typical indoor pollutant for decades. To evaluate the current state-of-the-art in formaldehyde research and to identify the plethora of regulated and unregulated formaldehyde sources in indoor and outdoor spaces, an extensive literature search was carried out. The acquired data were analyzed with the aid of Monte-Carlo methods to calculate realistic formaldehyde concentration profiles and exposure scenarios under consideration of aging, source/sink behavior and diffusion effects. Average concentrations of formaldehyde are within 20–30 µg/m³ for European households under residential-typical conditions. The assumption of an average air exchange rate of 0.5 h^−1^ is also plausible. Formaldehyde emission rates of materials and products for indoor use are widely spread and range from non-detectable to > 1000 µg/h. However, processes like combustion, cleaning activities, operation of air purifiers and indoor chemistry were identified as temporary but relevant formaldehyde sources, which might cause high peak concentrations.

**Specifications table**

**Table:** 

Subject area	Environmental Sciences
More specific subject area	Indoor Air
Type of data	Indoor air concentrations and material emission rates
How data was acquired	Survey and evaluation of the current literature
Data format	As taken from the cited references
Experimental factors	If necessary, data were converted from ppb to µg/m³ and vice versa. Chamber concentrations were converted into area specific and unit specific emission rates.
Experimental features	A literature survey was performed to collect published data about formaldehyde emissions from building materials and consumer products for indoor use in different databases.
Data source location	The data were taken from different sources (see cited references)
Data accessibility	All data can be assessed via the cited references.
Related research article	This article provides the scientific basis for the research paper: T. Salthammer (2019) Formaldehyde sources, formaldehyde concentrations and air exchange rates in European housings, Building and Environment, accepted for publication.

**Value of the data**

• This work was carried out to gather representative data in order to calculate realistic distributions of indoor related formaldehyde emission rates and formaldehyde concentrations in Europe.
• Data concerning formaldehyde concentrations in indoor and outdoor air, temporary and permanent sources, as well as data on air exchange, were collected for the European region.
• Material aging, source/sink behavior and diffusion effects were also considered.
• The data can be used to estimate human exposure to formaldehyde in the indoor environment under real-life conditions.

## 1 Data

An evaluation of potential formaldehyde sources, formaldehyde concentrations and air exchange rates is provided. A multitude of different permanent and temporary formaldehyde emission sources were identified. In addition to the typical building products, these also include chemical reactions occurring in indoor spaces, infiltrated outdoor air, combustion processes of all kinds, the operation of equipment such as air purifiers and emissions from human activities such as sauna, cooking and cleaning. The data represent the living behavior and indoor conditions in European housings. This means that all evaluated and presented formaldehyde emission rates of building and consumer products refer to their availability on the European market. Indoor and outdoor formaldehyde concentrations outside of Europe are not discussed.

## 2 Experimental design, materials, and methods

A literature survey was performed to collect published data about formaldehyde emissions from building materials and consumer products for indoor use in different databases:

CAS SciFinder (SF) http://www.cas.org/products/scifinder.

Web of Science (WoS) https://apps.webofknowledge.com.

SCOPUS (SCO) https://www.scopus.com/home.uri.

PubMed (PM) https://www.ncbi.nlm.nih.gov/pubmed/.

Google Scholar https://scholar.google.de/.

Keywords were chosen in a way that the number of hits was reduced sequentially until all papers published from 1990 on were extracted which contained data on emission rates from products used in indoor environments. Papers containing chamber concentrations from which emission rates could be calculated were considered as well. For this procedure, keywords describing products of relevance were identified in advance, such as textile, wood, particleboard, fibreboard/fibreboard, OSB, laminate, carpet, flooring, paper, adhesive, ceiling, foil, gypsum, insulation, sealant, furniture, paint, varnish, lacquer, film, tile, wallpaper, building material, construction material.

The sequential extraction of papers from the databases was performed as follows: At the beginning, all entries with the keyword “formaldehyde” were compiled. The SF database delivered approximately 267,500 hits, the WoS database approximately 43,750. The number of hits then was reduced by specification with the keyword emission, by excluding patents and setting a time limit for the year of publication from 1990 on. Further, only English, German, French, Italian or Spanish written papers were chosen. This procedure gave app. 5250 hits for SF and app. 3100 hits for WoS. From these, papers were taken with the keywords emission rate combined with the product specifications listed above. As a result, app. 570 papers from SF and app. 300 from WoS were identified. As a next step, all publications which could be excluded to be relevant because the title did not comply with the subject were removed. The same procedure was done with the remaining ones by checking the abstracts. Moreover, all reports and publications representing biased data were removed. Consequently, the report and database by Hofmann and Plieninger [1] was not considered. Finally, together with some studies from the WKI fundus including entries in the WKI owned sample database ERAD, 165 papers were collected in an EndNote database and closer investigated for relevant data. In case where concentrations (C) are given together with air exchange rates (ACH) and loading factors (L) (in case of area specific sources), area specific emission rates (SER_A_) and unit specific emission rates (SER_u_) were calculated. Reports and journals not being covered by scientific databases (e.g. HK Holz- und Möbelindustrie, Holztechnologie, Holz-Zentralblatt, etc…) were searched separately.

For each product or scenario the available data were summarized and, if possible, percentiles (10−P, 25−P, 50−P, 75−P, 90−P) were calculated. Then an appropriate function (normal, log-normal or a combination of both), which represents these percentiles best, was determined by use of a least-squares algorithm [2]. Finally, a stochastic Monte-Carlo approach was applied to calculate probability distributions from pseudo-random numbers with 100,000 runs per calculation. Ranges (uniform) are provided if the derivation of a statistical function was not possible.

### 2.1 Units

Many different units can be found in the international literature for the concentration of formaldehyde in air. In the following, only mass-related units will be used for the comparison of concentrations and emission rates. For the conversion of volume-related units (ppb and ppm) into mass-related units (µg/m³ and mg/m³) according to Eq. (1), a pressure of 1013 mbar (101,300 Pa), a temperature of 23 °C (293 K) and M(HCHO) = 30.03 g/mol will be assumed.

(1) p∙V=n∙R∙T

Therefore, 1 ppb = 1.24 µg/m³, 1 µg/m³ = 0.81 ppb, 0.1 ppm = 0.124 mg/m³ and 100 µg/m³ = 80.6 ppb.

In comparison to the thermodynamic standard chamber temperature of 25 °C (298 K) and a temperature of 20 °C (293 K) there is a marginal difference in the conversion factor (1.24 vs. 1.25 and 1.23), which will be neglected in the discussion.

### 2.2 Statistical software

The scientific software OriginPro 2016G (OriginLab Corporation, Northhampton, USA) was applied. The LabTalk script was used to calculate probability distributions.

ORIGIN LabTalk script representing a log-normal distribution: exp[normal(*N*)*σ+*µ*].

N is the number of calculated random variables, σ is the arithmetic standard deviation with *σ*_g_ = exp(*σ*), *µ* is the arithmetic mean with GM = exp(µ). GM is the geometric mean and *σ*_g_ is the geometric standard deviation.

## 3 Air exchange rates

**Fig. 1:**
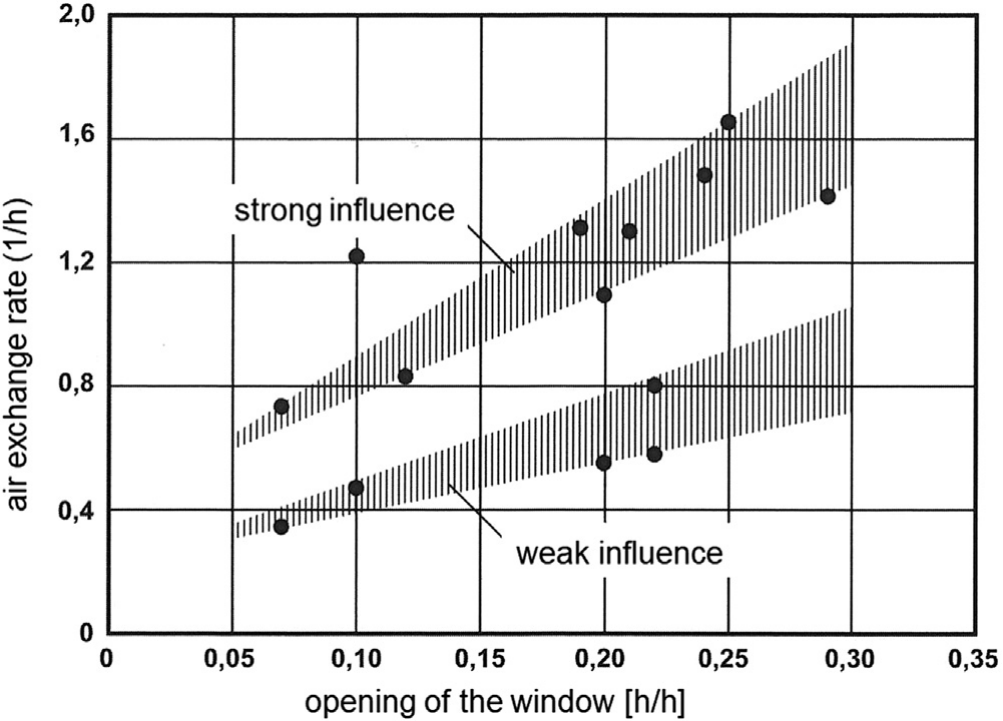
Average air exchange rates for manually ventilated houses (town houses and twin houses) in dependence of window opening. The figure was adapted with permission from Reiß and Ehrhorn [9], Copyright: Fraunhofer IRB-Verlag, all rights reserved.

**Fig. 2:**
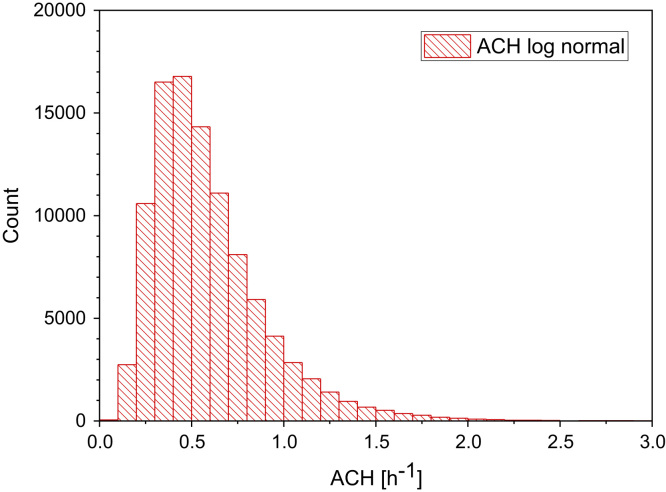
Monte-Carlo simulation of a log-normal distribution of air exchange rates (ACH) with 100,000 runs and a statistical interval of Δ(ACH) = 0.1 h^−1^. The statistical parameters are as follows: 25−P = 0.40 h^−1^, 50−P (median) = 0.52 h^−1^, 75−P = 0.68 h^−1^, GM = 0.52 h^−1^ and *σ*_g_ = 1.49 h^−1^. ORIGIN LabTalk: exp[(normal(100,000)*0.4+0.1)−0.75].

Table 1, Table 2 and Fig. 1, Fig. 2.

**Table 1:** Measured air exchange rates in different types of residential buildings in Europe and in the U.S.

**ACH [h**^**−1**^**]**	**Statistics**	**Condition**	**Reference**
0.60	Median	Conventional houses (Sweden)	Langer et al. [3]
0.68	Median	Passive houses (Sweden)	Langer et al. [3]
0.44	Median	Dwellings (France)	Langer et al. [4]
0.35	Median	Residences (U.S.)	Du et al. [5]
1.15	Median	Residences, basement (U.S.)	Du et al. [5]
0.08 – 0.69	Range	Low energy buildings (Lithuania)	Kaunelienė et al. [6]
0.43	Median	Renovated	Földváry et al. [7]
0.45	Median	Renovated	Földváry et al. [7]
0.4	Median	All dwellings, night-time, heating season (France)	Derbez et al. [8]
0.5	Median	All dwellings, night-time, non-heating season (France)	Derbez et al. [8]

**Table 2:** Influence of window opening on the average air exchange rates in housings [9].

**Window opening [h/h]**	**ACH [h**^**−1**^**]**	**Remarks**
0.05	0.35–0.6	Heating period
0.30	0.9–1.7	Heating period

## 4 Formaldehyde in ambient air

Table 3 and Fig. 3.

**Table 3:** Formaldehyde concentrations in outdoor air as determined in different international studies (GM = geometric mean). For better comparison, mass related data were converted to ppb and are marked with.^a^

**Location**	**C**_**HCHO**_**[ppb]**	**Comments**	**Reference**
Rural European sites	0.4–5.5	Range	Solberg et al. [10]
Kuopio, Finland	35/55	Maximum	Solberg et al. [10]
	1.0–2.2	Background	
Uppsala, Sweden	1.1^a^	GM	Sakai et al. [11]
Milan, Italy	1.5–13	Range	Hak et al. [12]
Rome, Italy	1.0–5.7^a^	Range	Santarsiero and Fuselli [13]
	2.0^a^	Median	
Athens, Greece	0.04–31.6^a^	Range	Bakeas et al. [14]
	12.9^a^	Median	
Barcelona, Spain	3.1–4.1	Range	Gallego et al. [15]
European cities	0.3–4.0	Range	Bruinen de Bruin et al. [16]

a Converted to “ppb.”

**Fig. 3:**
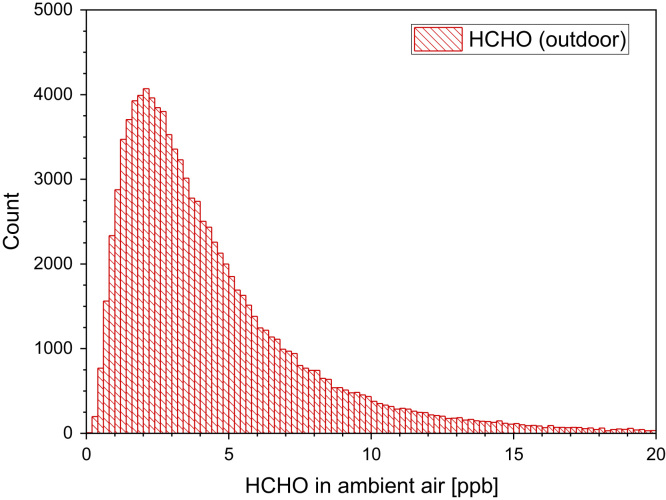
Monte-Carlo simulation of a log-normal distribution of formaldehyde outdoor concentrations with 100,000 runs and a statistical interval of Δ(HCHO) = 0.2 ppb. The statistical parameters are as follows: 25−P = 2.10 ppb, 50−P (median) = 3.49 ppb, 75−P = 5.78 ppb, GM = 3.49 ppb and σ_g_ = 2.11 ppb. ORIGIN LabTalk: exp[normal(100,000)*0.75+1.25].

## 5 Formaldehyde concentrations in indoor air under living conditions

**Fig. 4:**
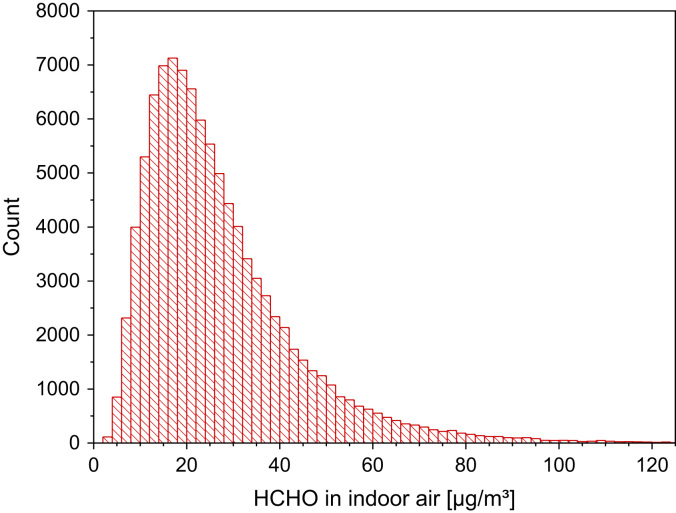
Monte-Carlo simulation of a log-normal distribution of formaldehyde concentrations under normal living conditions in European homes with 100,000 runs and a statistical interval of Δ(HCHO) = 2 µg/m³. The statistical parameters are as follows: 25−P = 15.7 µg/m³, 50−P (median) = 23.1 µg/m³, 75−P = 34.0 µg/m³, 95−P = 59.4 µg/m³, GM = 23.1 µg/m³ and σ_g_ = 1.78 µg/m³. ORIGIN LabTalk: exp[normal(100,000)*0.575+3.14].

Table 4, Table 5 and Fig. 4.

**Table 4:** Formaldehyde concentrations in indoor air as determined in different international studies. *N* is the number of measurements.

**Country**	***N***	**GM [µg/m³]**	**50−P [µg/m³]**	**75−P [µg/m³]**	**95−P [µg/m³]**	**Reference**
Germany	586	23.3	23.5		47.7	Umweltbundesamt [17]
France^a^	143		26.7			Marchand et al. [18]
France^b^	143		30.9			Marchand et al. [18]
France	554	19.5	19.7	29		Langer et al. [4]
Sweden^c^	20		11.1			Langer et al. [3]
Sweden^d^	21		15.7			Langer et al. [3]
Sweden^e^	294	16.0	17.0			Langer and Bekö [19]
England	876	22.2	24.0	35.2	61.2	Raw et al. [20]
Spain^a^	10		22.5	*(31)*^f^		Rovira et al. [21]
Spain^b^	10		27.3	*(38)*^f^		Rovira et al. [21]
Italy	40		10.6			Santarsiero and Fuselli [13]
Italy	59		14.2			Lovreglio et al. [22]
Lithuania^g^	11		30.8	40.5		Kaunelienė et al. [6]
Denmark^h^	20		40			Kolarik et al. [23]
Slovakia^i^	20	30	30			Földváry et al. [7]
Slovakia^i^	20	41	42			Földváry et al. [7]
France^j^	65		13.8	19.1		Derbez et al. [8]
France^k^	65		19.4	25.4		Derbez et al. [8]

a Living room.

b Bedroom.

c New passive houses.

d New conventional houses.

e Housing stock.

f Estimated from boxplot.

g Low energy houses.

h New Danish buildings.

i Renovated building.

j Master bedroom, heating season.

k Master bedroom, non-heating season.

**Table 5:** Formaldehyde indoor and outdoor concentration (arithmetic mean and standard deviation) from the AIRMEX study (see Bruinen de Bruin et al. [16] for details). *N* is the number of measurements.

**Location**	**Outdoor [ppb]**			**Indoor (public build.) [ppb]**			**Indoor (homes) [ppb]**		
	***N***	***µ***	***σ***	***N***	***µ***	***σ***	***N***	***µ***	***σ***
Brussels	3	2.7	0.5	16	13.9	5.6	3	19.5	3.0
Budapest	7	2.1	0.3	12	18.2	6.8	7	24.4	9.2
Leipzig	14	2.2	0.5	28	22.9	10.4	7	28.6	13.4
Helsinki	5	2.1	0.3	11	19.7	9.8	12	28.8	9.3
Arnhem	3	2.0	0.4	5	17.7	10.4	5	30.7	17.8
Athens	10	3.2	1.3	20	20.5	8.8	14	24.1	12.9
Catania	12	3.7	0.8	17	14.7	5.0			
Dublin	6	0.4	0.2	11	17.5	13.3	7	14.4	4.9
Nijmegen	2	2.4	0.1	4	19.5	6.8	2	30.1	24.2
Thessaloniki	8	4.9	1.4	7	20.6	8.3

## 6 Formaldehyde concentrations in indoor air under steady-state conditions

Table 6 and Fig. 5.

**Table 6:** Formaldehyde steady-state concentrations in living rooms and bedrooms of Austrian dwellings (see Tappler et al. [24] and Wallner et al. [25] for details). *N* is the number of measurements.

**N**	**50−P [µg/m³]**	**95−P [µg/m³]**	**Ventilation system**	**Campaign**
62 (test group)	27	53	yes	First
61 (test group)	22	46	yes	Second
61 (control group)	40	67	no	First
59 (control group)	31	59	no	Second

**Fig. 5:**
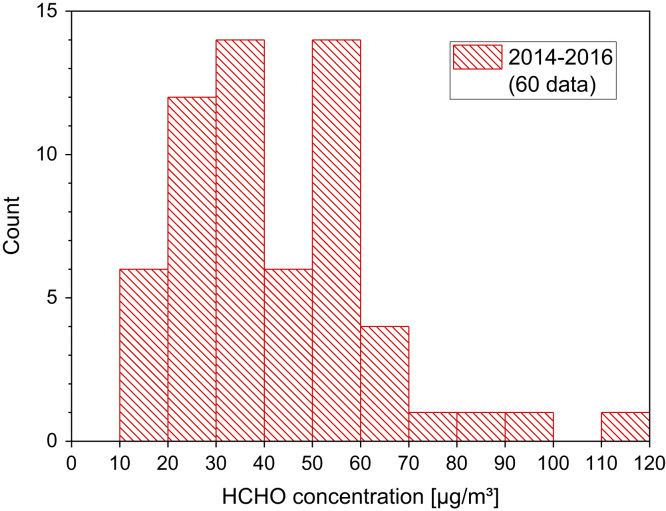
Distribution of formaldehyde steady-state concentrations in newly built prefabricated houses in Germany (N = 60, AM = 41.8 µg/m³, GM = 37.2 µg/m³, 25−P = 27 µg/m³, 50−P (median) = 38 µg/m³; 75−P = 50.8 µg/m³). Data by Courtesy of Bundesverband Deutscher Fertigbau e. V. (2017).

## 7 Formaldehyde concentrations in special indoor environments – sauna cabins

Table 7.

**Table 7:** Formaldehyde concentrations in sauna cabins according to Wegscheider et al. [26] (see this reference for experimental details). The synonyms should be interpreted as follows: “cold”: before operation; “hot”: during operation; “Eucalyptus, Birch, Citrus, Mint, Herbs, Menthol”: type of essence.

**Sauna**	**HCHO cold [mg/m³]**	**HCHO hot [mg/m³]**	**HCHO Eucalyptus [mg/m³]**	**HCHO Birch [mg/m³]**	**HCHO Citrus [mg/m³]**	**HCHO Mint [mg/m³]**	**HCHO Herbs [mg/m³]**	**HCHO Menthol [mg/m³]**
1	0.01	0.18	0.79	1.1				
2	0.01	0.25				0.95	2	
3	0.02	0.37						0.56
4		0.08	0.11					
4		0.1			0.22			
4		0.12	0.14					
4		0.12			0.19			
4		0.21				0.21		
5	0.04	0.28	0.32					
5		0.15			0.23			
5		0.15	0.16					
5		0.14			0.33			
5		0.17				0.17		
5		0.03				0.03		
6	0.03	0.06	0.05		0.04			
6			0.1		0.16	0.07		
7	0.01	0.13			0.25			
7					0.56		0.35	
7		0.05			0.55			
7					2.1		0.7	
7					0.47			
7	0.01	0.08			0.48		0.86	
7					0.6		0.66	
7					2			

## 8 Formaldehyde from indoor chemistry

**Fig. 6:**
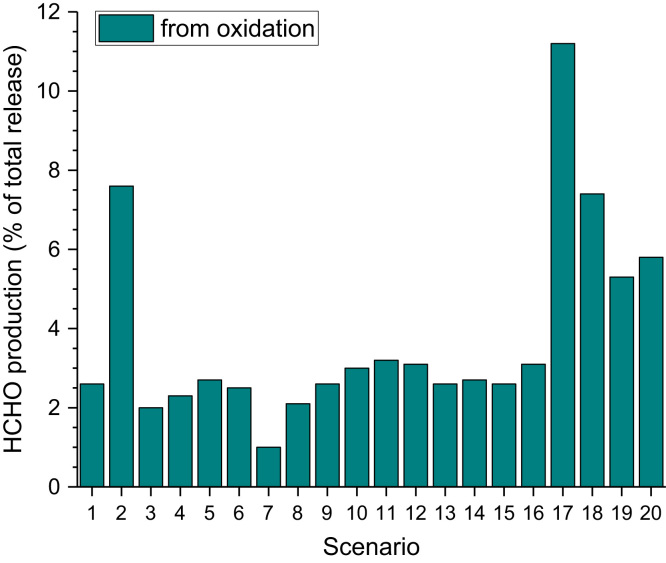
Percentage of production of formaldehyde for 20 different indoor scenarios (the data are taken from Mendez et al. [36]).

**Fig. 7:**
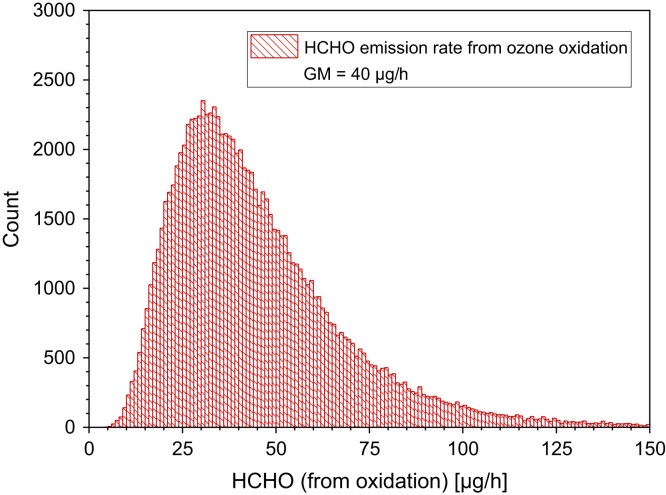
Monte-Carlo simulation of a log-normal distribution of formaldehyde emission rates from indoor chemistry with 100,000 runs and a statistical interval of Δ(HCHO) = 1 µg/h. The statistical parameters are as follows: 25−P = 28.62 µg/h, 50−P (median) = 40.05 µg/h, 75−P = 56.18 µg/h, GM = 40.05 µg/h and σ_g_ = 1.65 µg/h. ORIGIN LabTalk: exp[normal(100,000)*0.5+3.69].

Table 8 and Fig. 6, Fig. 7.

**Table 8:** Formaldehyde concentrations as determined in test chamber experiments in the presence of ozone. See references for experimental details. For better comparison, mass related data were converted to ppb and are marked with “^a^”.

**Material**	**C**_**HCHO**_**[ppb]**	**Comments**	**Reference**
Carpet	1.3 – 8.1	28 – 44 ppb O_3_	Weschler et al. [27]
	0.5 – 4.6	no O_3_	
Gas phase	74.4 – 407.2^a^	266 – 770 µg/m³ limonene	Zhang et al. [28]
		53 – 298 µg/m³ O_3_	
Gas phase	19.2 – 28.8^a^	310 – 1694 µg/m³ VCH	Zhang et al. [28]
		177 - 293 µg/m³ O_3_	
Latex paint	*Emission rates presented, see reference*		Reiss et al. [29]
Different materials	<4 – 112^a^	40 – 80 ppb O_3_	Moriske et al. [30]
Carpet	*Emission rates presented, see reference*		Morrison and Nazaroff [31]
Carpet	*Emission rates presented, see reference*		Abbass et al. [32]
Air freshener	11.2 - 23.7	60 ppb O_3_	Singer et al. [33]
Painted wooden board	104^a^	50 ppb O_3_ (max.)	Huang et al. [34]
	40^a^	50 ppb O_3_ (24 h)	
Cleaning agent	2.4^a^	5 ppb O_3_	Norgaard et al. [35]
	8.8^a^	50 ppb O_3_	

a Converted to “ppb”

## 9 Formaldehyde from the burning of candles

In a so far unpublished WKI study by Wensing a formaldehyde emission rate of 96 µg/g was measured. With a mass loss of 4 g/h this can be converted to a time related value of 384 µg/h. Derudi et al. [39] measured formaldehyde emission rates between 2 µg/g and 3 µg/g from scented candles but did not determine the mass loss.

**Fig. 8:**
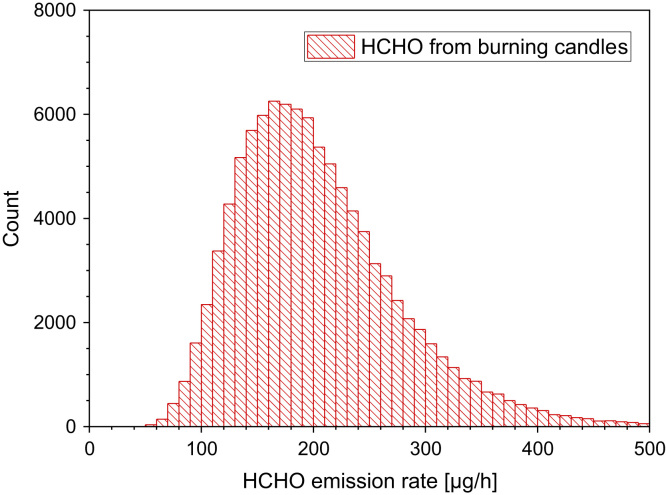
Monte-Carlo simulation of a log-normal distribution of formaldehyde emission rates from the burning of candles with 100,000 runs and a statistical interval of Δ(HCHO) = 10 µg/h. The statistical parameters are as follows: 25−P = 151.8 µg/h, 50−P (median) = 192.4 µg/h, 75−P = 243.5 µg/h, GM = 192.5 µg/h and σ_g_ = 1.42 µg/h. ORIGIN LabTalk: exp[normal(100,000)*0.35+5.26].

Petry et al. [40] also studied formaldehyde emission rates from fragranced and unfragranced candles. The results are as follows: 137.9 µg/h, 235.3 µg/h, 73.0 µg/h, 283.9 µg/h, 372.2 µg/h, 316.5 µg/h, 19.6 µg/h, 234.0 µg/h, 289.0 µg/h, 280.0 µg/h, <25.7 µg/h (Table 9 and Fig. 8).

**Table 9:** Emission factors of formaldehyde released from scented burning candles (Ahn et al. [37]; Kim et al. [38]).

**Type of candle**	**Mass loss [g/min]**	**Emission rate [µg/g]**	**Emission rate [µg/h]**
Clean Cotton	0.068	36.9	151
Floral	0.041	2.67	7
Kiwi melon	0.074	8.85	39
Strawberry	0.054	95.7	310
Vanilla	0.078	0.59	3
Plain	0.082	27.6	136

## 10 Formaldehyde from incense burning

Lee and Wang [42] studied the release of formaldehyde from 10 types of incense sticks in an 18.26 m³ stainless-steel chamber at *T* = 23 °C, RH = 5=% and ACH = 0.5 h^−1^. The average burn time was between 25 min and 51 min. The chamber concentrations ranged from approx. 20 µg/m³ to 300 µg/m³. Mass related formaldehyde emission rates ranged from approx. 400 µg/g to 1700 µg/g (Table 10).

**Table 10:** Formaldehyde concentrations in a test house from the burning of incense sticks and cones. The data are taken from Maupetit and Squinazi [41]. Abbreviations P1–P4 refer to the nomenclature in the paper.

**Type of incense**	**Max [µg/m³]**	**50−P [µg/m³]**
Stick (P1)	37.1	9.8
Stick (P2)	39.3	6.4
Stick (P3)	38.2	3.8
Stick (P4)	47.5	2.9
Cone (P1)	51.0	39.3
Cone (P2)	57.6	28.5
Cone (P3)	43.8	9.7
Cone (P4)	51.0	10.0

Maupetit and Squinazi [41] studied the release of formaldehyde from incense sticks and incense cones in a 32.3 m³ test house at *T* = 20 °C and ACH = 0.6 h^−1^. The burnt mass of the sticks was between 0.16 g and 1.25 g with a 50−P value (median) of 0.32 g. The duration of combustion was between 15 min and 64 min with a 50−P value (median) of 29 min. The burnt mass of the cones was between 0.39 g and 0.90 g with a 50−P value (median) of 0.49 g. The duration of combustion was between 10 min and 25 min with a 50−P value (median) of 17 min.

## 11 Formaldehyde from the consumption of conventional and electronic cigarettes

As in the case of other combustion sources, the emission rate is often presented in the unit μg/cig (mass emitted per cigarette burnt) (Table 11, Table 12). A summary of formaldehyde emissions from conventional cigarettes can be found in the review by Salthammer et al. [113].

**Table 11:** Indoor air concentrations (µg/m³) of formaldehyde measured during a 2 hour use of e-cigarettes containing different liquids with (+) or without (−) nicotine in a 45 m³ room at ACH = 0.56 h^−1^. The data were taken from Schober et al. [43].

**Compound**	**No vaping [µg/m³]**	**Liquid 1 [µg/m³]**		**Liquid 2 [µg/m³]**		**Liquid 3 [µg/m³]**	
**formaldehyde**		**(−)**	**(+)**	**(−)**	**(+)**	**(−)**	**(+)**
	25.0	24.0	28.0	27.0	55.0	28.0	21.0

**Table 12:** Duplicate determinations of analyte concentrations in the vapor generated by an electronic cigarette device filled with an e-liquid in dependence of the battery setting [44].

**Measurement**	**Battery setting**			
	**3.3 V**	**3.8 V**	**4.3 V**	**4.8 V**
Measurement #1 (µg/puff)	46	45.9	34.9	93
Measurement #2 (µg/puff)	61	45.9	35.0	101

## 12 Formaldehyde from cooking and cooking related activities

**Fig. 9:**
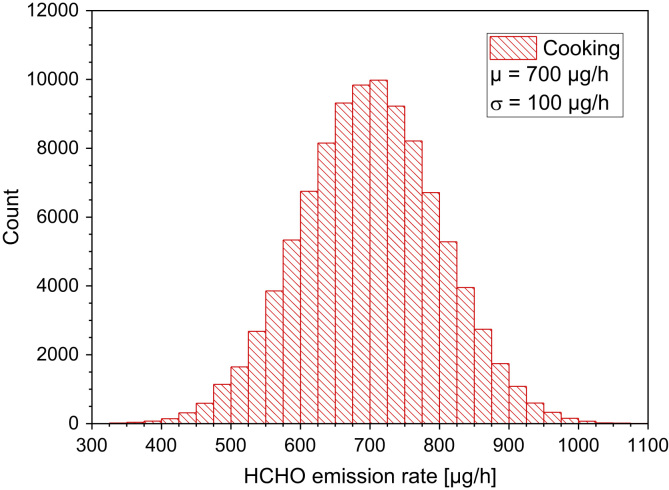
It is difficult to calculate a realistic distribution of formaldehyde emission rates on the basis of the available data set. The highest concentrations from Table 13 refer to oven cleaning rather than the cooking process itself. When taking into account the available data, a normal distribution was calculated with a mean value *µ* = 700 µg/h and a standard deviation σ = 100 µg/h. It should, however, be mentioned that the assumed normal distribution only provides a very rough estimation of a realistic cooking scenario. ORIGIN LabTalk: normal(100,000)*100+700.

Logue et al. [46] studied pollutant exposures from natural gas cooking burners by use of models. For the winter period one week time averaged formaldehyde concentrations were 1 ppb (median) and 13 ppb (95−P), respectively. The highest one hour average concentrations were approximately 13 ppb (median, summer), 111 ppb (95−P, summer), 19 ppb (median, winter) and 158 ppb (95−P, winter), respectively (Table 13 and Fig. 9).

**Table 13:** Concentrations of formaldehyde during selected cooking tests [45].

**Activity**	**Oven**	**Condition**	**Kitchen [µg/m³]**	**Outdoor [µg/m³]**
Oven cleaning	Gas	Standard	417.3	2.7
Broil fish	Gas	Standard	129.3	1.5
Oven cleaning	Electric	Standard	224.5	0.8
Broil fish	Electric	Standard	129.4	0.4
Pork roast	Gas	Aluminum pan	49.1	1.0
Pork roast	Gas	Exhaust vent.	36.5	1.1

Peng et al. [47] studied effects of cooking method, cooking oil, and food type on aldehyde emissions in cooking oil fumes. The formaldehyde concentrations in the oil fumes were between 4 µg/m³ and 27 µg/m³, depending on the cooking oil (palm rapeseed, sunflower, soybean) and the cooking method (pan-frying, deep-frying, stir-frying). The formation and emission of formaldehyde and other organic compounds from the heating of fatty acids and fatty acid esters was reviewed by Abdullahi et al. [48]. Formaldehyde is also formed by Strecker degradation in Maillard systems [49].

Bednarek et al. [50] performed a study on human exposure to air pollutants during a dinner. Seven adults volunteered in a 55 m³ room at ACH = 0.29 h^−1^. During the cooking phase (indoor barbecue) the formaldehyde concentration increased from 23 µg/m³ to 58 µg/m³ within two hours. The consumption of 33 cigarettes led to a further increase of the formaldehyde concentration to 154 µg/m³.

## 13 Formaldehyde from ethanol fireplaces

Guillaume et al. [52] also measured high formaldehyde concentrations between 0.4 mg/m³ and 0.9 mg/m³ in the exhaust gas of four decorative ethanol fireplaces (Table 14). Höllbacher et al. [53] studied a single device and measured 62 µg/m³ formaldehyde in a model room. Formaldehyde sources from combustion (candles, ethanol fireplaces, mosquito coils, etc.) were reviewed by Szulejko and Kim [54].

**Table 14:** Experimental conditions, mean formaldehyde concentrations and calculated mean emission rates during the burning phase of four fireplaces (O1–O4) with different types of fuel (see Schripp et al. [51] for more details).

**Type of fuel**	**V [m³]**	**ACH [h**^**−1**^**]**	**t**_**burning**_**[h]**	**C**_**HCHO**_**(mean) [ppb]**	**C**_**HCHO**_**(max) [ppb]**	**Emission rate [µg/h]**
O1 – ethanol (98%)	48	0.69	1.70	131	210	5380
O1 – ethanol (94%)	48	0.69	1.40	259	456	10,637
O1 – ethanol (94%)	48	0.69	1.45	17	35	698
O3 – gel-type	48	0.91	3.10	56	129	3033
O3 – gel-type	48	0.91	2.60	60	177	3249
O3 – gel-type	48	0.91	3.20	54	202	2925
O4 – gel-type	48	0.43	2.50	39	67	998
O3 – gel-type	48	0.91	2.80	36	47	1949

## 14 Formaldehyde emission from miscellaneous products

Table 15, Table 16.

**Table 15:** Formaldehyde maximum concentration levels (24 h mean) for calculated scenarios from the EPHECT project (see Dimitroulopoulou et al. [55], [56] and Trantallidi et al. [57] for details).

**Product**	**ACH [h**^**−1**^**]**	**V [m³]**	**C**_**HCHO**_**[ppb]**	**Remark**
All−Purpose cleaning agent	0.1	24	< 1	Spray
All−Purpose cleaning agent	0.1	17	1	Liquid
Kitchen cleaning agent	0.35	24	< 1	Liquid
Floor cleaning agent	0.35	24	6	Liquid
Floor cleaning agent	0.1	17	30	Liquid
Floor cleaning agent	0.3	24	3	Liquid
Floor cleaning agent	0.1	24	6	Liquid
Furniture polish	0.1	32	1	Spray
Floor polish	0.1	45	< 1	Liquid
Electric air freshener	0.3	24	7	Liquid
Electric air freshener	0.1	24	19	Liquid
Perfume	0.1	17	< 1	Spray−Pump

**Table 16:** Data from the study by Lefebvre et al. [58]. Subject blanks (bathroom with study subject), range of maximum air concentrations of formaldehyde in the bathroom after product application and mean bathroom concentrations. The conditions were as follows: *V* = 9.4 m³, *T* = 23 °C, RH = 30–50%, ACH = 5 h^−1^.

**Product**	**Subject blank (mean) [µg/m³]**	**Peak conc. (range) [µg/m³]**	**Room conc. (mean) [µg/m³]**
Facial moisturizer	1.9–3.3	3.1–14.4	3.3
Body lotion	1.5–4.1	5.4–17.8	6.2
Foundation	1.5–2.4	2.9–4.8	2.8
Shower gel	1.2–3.0	0.9–4.7	2.7
Shampoo	1.5–3.3	1.9–5.3	2.5
Deodorant	2.1–3.8	1.9–5.3	2.6
Hair conditioner	1.7–3.8	3.5–8.7	4.5
Hair styling gel	1.7–6.0	3.3–10.9	2.7

## 15 Formaldehyde from wood combustion

Table 17, Table 18.

**Table 17:** Formaldehyde emission factors (EF) from residential wood combustion. Note: these emission factors refer to the formation of formaldehyde from the combustion process. They do not refer to the release of formaldehyde into the indoor environment.

**Biomass**	**Appliance**	**EF**_**HCHO**_**[mg/kg]**	**Reference**
Maritime pine	Wood stove	653	Cerqueira et al. [59]
Eucalyptus	Wood stove	1038	Cerqueira et al. [59]
Cork oak	Wood stove	1080	Cerqueira et al. [59]
Holm oak	Wood stove	988	Cerqueira et al. [59]
Pyrenean oak	Wood stove	1772	Cerqueira et al. [59]
Softwood	Fireplace	113	McDonald et al. [60]
Hardwood	Fireplace	178	McDonald et al. [60]
Hardwood	Wood stove	246	McDonald et al. [60]
Pine	Fireplace	1165	Schauer et al. [61]
Oak	Fireplace	759	Schauer et al. [61]
Eucalyptus	Fireplace	599	Schauer et al. [61]
Birch	Wood stove	422	Hedberg et al. [62]

**Table 18:** Formaldehyde concentrations in private homes before and during operation of wood burning fireplace ovens (see Salthammer et al. [63] for details).

**Oven**	**C**_**HCHO**_**[ppb] before operation**	**C**_**HCHO**_**[ppb] during operation**
1	12	18
2	14	18
3	16	55
4	16	34
5	10	16
6	19	20
7	10	19

Lévesque et al. [64] investigated 31 Canadian homes and found no difference in the HCHO concentrations in relation to the sampling location nor in relation to whether a combustion appliance was present or not.

## 16 Formaldehyde from air cleaning devices and paints

Sidheswaran et al. [67] demonstrated that at room temperature and 80% RH the indoor formaldehyde concentrations increased from 9–12 μg/m³ to 12–20 μg/m³ when synthetic filters were replaced with fiberglass filtration media in the HVAC units (Table 19, Table 20).

**Table 19:** Initial and final steady-state formaldehyde concentrations in a 20 m³ chamber under different conditions during operation of PCO filters (see Destaillats et al. [65] for details).

**UV-type/experiment**	**UVC/2**	**UVC/3**	**UVC/4**	**UVA/5**	**UVA/6**
C_initial_ [µg/m³]	30 ± 1	20 ± 1	29 ± 5	27 ± 4	29 ± 1
C_steady-state_ [µg/m³]	44 ± 1	33 ± 1	22 ± 3	18 ± 1	11 ± 1

**Table 20:** Steady-state formaldehyde concentrations in a 14.75 m³ stainless-steel chamber in absence and presence of air freshener and operation of an air cleaning device (see Waring et al. [66] for details).

**Formaldehyde steady-state concentration [µg/m³]**			
**Background**	**Air cleaner**	**Air cleaner + air freshener**	**Air freshener**
17.6 ± 2.8	19.3 ± 2.8	49.3 ± 3.9	45.9 ± 2.7

More data on the release of formaldehyde from air cleaners and photocatalytic paints are available from Farhanian and Haghighat [68], Zhong et al. [69], Gunschera et al. [70], Ongwandee and Kruewan [71], Salthammer and Fuhrmann [72], Auvinen and Wirtanen [73] and Geiss et al. [74].

## 17 Formaldehyde from textiles

**Fig. 10:**
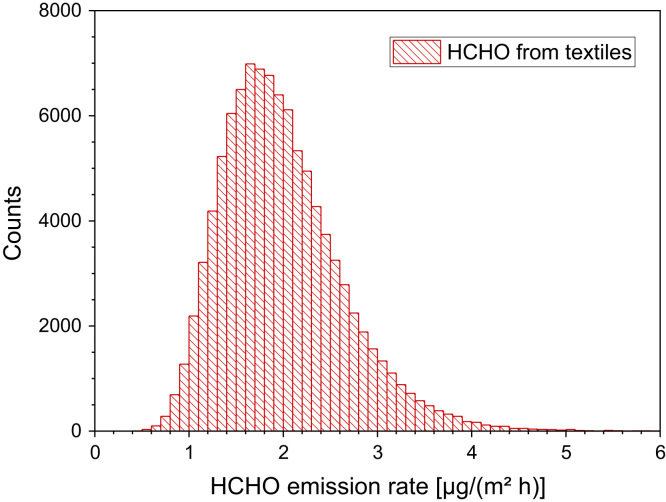
Monte-Carlo simulation of a log-normal distribution of formaldehyde emission rates from textiles with 100,000 runs and a statistical interval of Δ(HCHO) = 0.1 µg/(m² h). The statistical parameters are as follows: 25−P = 1.5 µg/(m² h), 50−P (median) = 1.9 µg/(m^2^ h), 75−P = 2.3 µg/(m² h), GM = 1.9 µg/(m² h) and σ_g_ = 1.38 µg/(m² h). ORIGIN LabTalk: exp[normal(100,000)*0.32 +0.642].

Table 21 and Fig. 10.

**Table 21:** Formaldehyde steady-state concentrations and emission rates from chamber experiments (*T* = 23 °C, RH = 45%, ACH = 1.0 h^−1^) and results from extraction analysis. The data were taken from Aldag et al. [75]).

**Sample**	**Material**	**Steady-state [ppb]**	**Emission rate [µg/(m² h)]**	**Emission rate [µg/(g h)]**	**Extraction [mg/kg]**
Curtain	100% cotton	4.3	5	0.15	
Curtain	100% polyester	1.3	2	0.12	
Curtain	100% polyacrylics	1.6	2	0.04	
Curtain	100% viscose	0.8	1	0.07	
Pants	100% cotton	< 0.4	< 0.4	< 0.01	11.0
T-shirt	100% cotton	2.3	3	0.07	17.7
Pants	100% linen	< 0.4	< 0.4	< 0.01	11.0
T-shirt	100% linen	2.9	4	0.07	10.1
Pants	100% polyester	2.4	3	0.05	24.8
T-shirt	100% polyester	< 0.4	< 0.4	< 0.01	5.7
Pants	100% polyamide	2.0	3	0.07	2.9
T-Shirt	100% polyamide	< 0.4	< 0.4	< 0.01	75.9
Shirt	55% cotton 45% polyester	0.6	1	0.03	4.3
Shirt	96% viscose 4% elastane	3.2	5	0.08	6.2

## 18 Formaldehyde from carpet

**Fig. 11:**
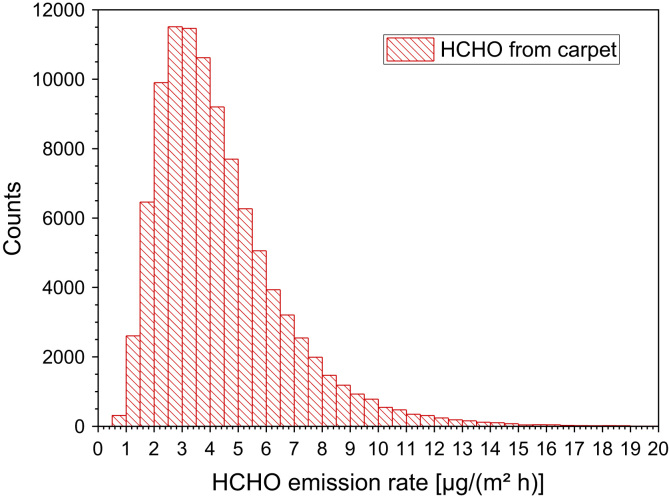
Monte-Carlo simulation of a log-normal distribution of formaldehyde emission rates from carpet with 100,000 runs and a statistical interval of Δ(HCHO) = 0.5 µg/(m² h). The statistical parameters are as follows: 25−P = 2.8 µg/(m² h), 50−P (median) = 3.9 µg/(m^2^ h), 75−P = 5.4 µg/(m² h), GM = 3.9 µg/(m² h) and *σ*_g_ = 1.65 µg/(m² h). ORIGIN LabTalk: exp[normal(100,000)*0.5+1.35].

Hodgson et al. [76] determined the area specific emission rates from at least four samples. In one case the emission rate could be quantified with SER_A_(24 h) = 57.2 µg/(m² h) and SER_A_(168 h) = 18.2 µg/(m² h). In all other experiments, the maximum formaldehyde concentrations in the chamber were 5 ppb or less (Fig. 11).

Morrison and Nazaroff [31] studied carpet for area specific emission rates of formaldehyde in test chambers at *T* = 23 °C and RH = 50%. In three cases the emission rates were between 9 µg/(m² h) and 15 µg/(m² h). In the other five cases, the emission rates were below 4 µg/(m² h).

In the work by Katsoyiannis et al. [77], the 72 h chamber concentrations obtained from three carpets in three different chambers were between 2.8 µg/m³ and 14 µg/m³. Under assumption of steady-state conditions the calculated area specific emission rates are between 3.5 µg/(m² h) and 17.5 µg/(m² h).

Abbass et al. [32] conducted tests with six types of new unused carpets using 52 l glass chambers at *T* = 21 °C, RH = 50%, ACH = 3 h^−1^ and *L* = 0.8 m²/m³. In the absence of ozone, the 24 h formaldehyde emission rates of five samples were between 10 µg/(m² h) and 16 µg/(m² h).

## 19 Formaldehyde from wallcoverings

**Fig. 12:**
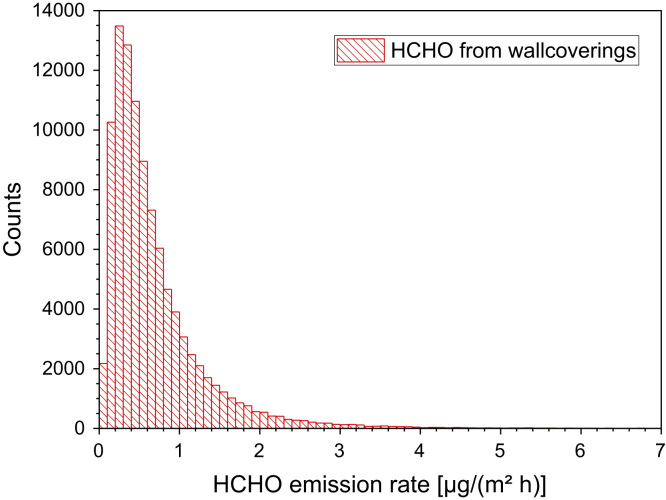
Monte-Carlo simulation of a log-normal distribution of formaldehyde emission rates from wallcoverings with 100,000 runs and a statistical interval of Δ(HCHO) = 0.1 µg/(m² h). The statistical parameters are as follows: 25−P = 0.3 µg/(m² h), 50−P (median) = 0.5 µg/(m^2^ h), 75−P = 0.9 µg/(m² h), GM = 0.5 µg/(m² h) and σ_g_ = 2.23 µg/(m² h). ORIGIN LabTalk: exp[normal(100,000) *0.8-0.6].

Fig. 12 and Table 22, Table 23.

**Table 22:** Calculated area specific emission rates for the release of formaldehyde from different types of wallcoverings (paper, textile, vinyl, acrylic) in the steady-state at *T* = 23 °C and RH = 45%. The experiments were performed in test chambers and by use of the WKI flask method between 1990 and 1992 (see Salthammer et al. [78]).

**No. of samples**	**SER**_**A**_**< 1 µg/(m² h)**	**SER**_**A**_**1 - 10 µg/(m² h)**	**SER**_**A**_**11 - 30 µg/(m² h)**	**SER**_**A**_**31 - 60 µg/(m² h)**
27	0	20	5	2

**Table 23:** Calculated area specific emission rates after 3 d and 28 d for the release of formaldehyde from wallcoverings at *T* = 23 °C and RH = 45%. The experiments were performed in test chambers between 2011 and 2016 (WKI data, unpublished).

**No. of samples**	**SER**_**A**_**< 1 µg/(m² h)**	**SER**_**A**_**1 - 10 µg/(m² h)**	**SER**_**A**_**11 - 30 µg/(m² h)**	**SER**_**A**_**31 - 60 µg/(m² h)**
144 (after 3 d)	107	28	6	3
97 (after 28 d)	89	7	1	0

## 20 Formaldehyde from surface coatings

**Fig. 13:**
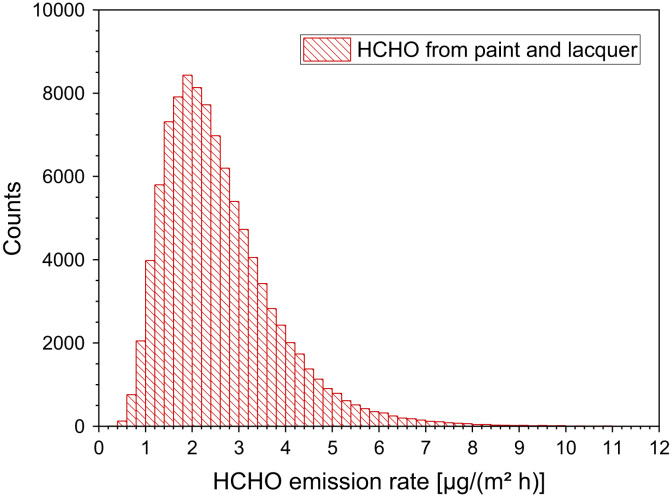
Monte-Carlo simulation of a log-normal distribution of formaldehyde emission rates from paint and lacquer with 100,000 runs and a statistical interval of Δ(HCHO) = 0.2 µg/(m² h). The statistical parameters are as follows: 25−P = 1.7 µg/(m² h), 50−P (median) = 2.3 µg/(m^2^ h), 75−P = 3.2 µg/(m² h), GM = 2.3 µg/(m² h) and σ_g_ = 1.56 µg/(m² h). ORIGIN LabTalk: exp[normal(100,000)*0.44–0.83].

Reiss et al. [29] measured emission rates between 0.05 µg/h and 3.45 µg/h with a median of 0.21 µg/h of 11 types of latex paint in a flow reactor. Chang et al. [81] studied the drying process of latex paint in a chamber at *T* = 23 °C, RH = 50%, ACH = 0.5 h^−1^ and *L* = 0.48 m²/m³. Within 50 h after application the formaldehyde chamber concentration of one paint was in the range of 0.5 mg/m³, while the chamber concentration of a different paint was 0.01 mg/m³. In a second study under identical chamber conditions, Chang et al. [82] followed the drying process of a freshly applied latex paint and measured a chamber concentration of about 0.1 mg/m³ after 300 h (Table 24, Table 25, Table 26 and Fig. 13).

**Table 24:** Emission rates of formaldehyde from so-called environmentally friendly paint on glass plates by use of the Field and Laboratory Emission Cell (FLEC). The data are taken from Horn et al. [79].

**Test sample no.**	**Formaldehyde SER**_**A**_**[µg/(m² h)]**					
	**0.01 d**	**1 d**	**9 d**	**37 d**	**42 d**	**85 d**
Paint 18^a^	3	2	0.6	10		
Paint 21^a^	9	2	5	8		4
Paint 22^a^	3	1	7		12	

a Nomenclature of paints by Horn et al. [79].

**Table 25:** Calculated area specific emission rates of formaldehyde from different paints and lacquers. The chamber concentrations and experimental conditions are taken from Horn et al. [80].

**Test sample no.**	**Formaldehyde SER**_**A**_**[µg/(m² h)]**	
	**10 d**	**28 d**
3587 (flooring paint)^a^	3	3
3463 (dispersion)^a^	5	5
3584 (dispersion)^a^	1	2
3586 (dispersion)^a^	5	2
3626 (dispersion)^a^	4	1
3690 (latex-dispersion)^a^	8	3

a Nomenclature of paints by Horn et al. [80].

**Table 26:** Area specific emission rates of formaldehyde from different types of lacquers applied on aluminum (see Schieweck and Salthammer [83] for experimental details). The measurements were carried out in test chambers by A. Schieweck within the framework of her PhD thesis.

**Test sample**	**Formaldehyde SER**_**A**_**[µg/(m² h)]**				
	**24 h**	**48 h**	**72 h**	**120 h**	**144 h**
2 K polyester^a^	1	2	< 1	< 1	
Epoxy^a^	< 1	< 1	< 1		
2 K polyurethane^a^	< 1	< 1	< 1		< 1
2 K polyurethane^a^	1	1	1		1
Cellulose nitrate^a^	1	1	< 1		< 1
2 K polyurethane^a^	< 1	< 1	< 1		< 1

a Nomenclature of samples by Schieweck (unpublished).

## 21 Formaldehyde from solid wood

**Fig. 14:**
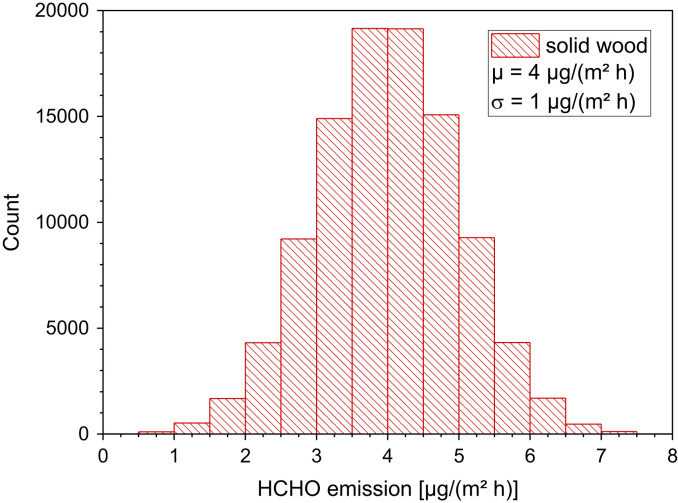
The available data did not allow the calculation of log-normally distributed emission rates. The Shapiro–Wilk test did not reject the hypothesis of normally distributed data on a 95% confidence level. When considering the small number of data, a conservative approach was applied to calculate a normal distribution with a mean value of 4 µg/(m² h) and a standard deviation of 1 µg/(m² h). ORIGIN LabTalk: normal(100,000)*1.0+4.0.

Table 27 and Fig. 14.

**Table 27:** Calculated area specific emission rates from solid wood.

**Wood type**	**SER**_**A**_**[µg/(m² h)]**	**Reference**
Oak	4	Risholm-Sundmann et al. [84]
Pine	5	Risholm-Sundmann et al. [84]
Beech	7	Böhm et al. [85]
Poplar	4	Böhm et al. [85]
Birch	4	Böhm et al. [85]
Oak	4	Böhm et al. [85]
Pine	5	Böhm et al. [85]
Spruce	6	Böhm et al. [85]
Beech	3	Meyer and Boehme [86]
Douglas fir	5	Meyer and Boehme [86]
Oak	4	Meyer and Boehme [86]
Spruce	4	Meyer and Boehme [86]
Pine	5	Meyer and Boehme [86]

## 22 Formaldehyde emission from raw wood-based materials

**Fig. 15:**
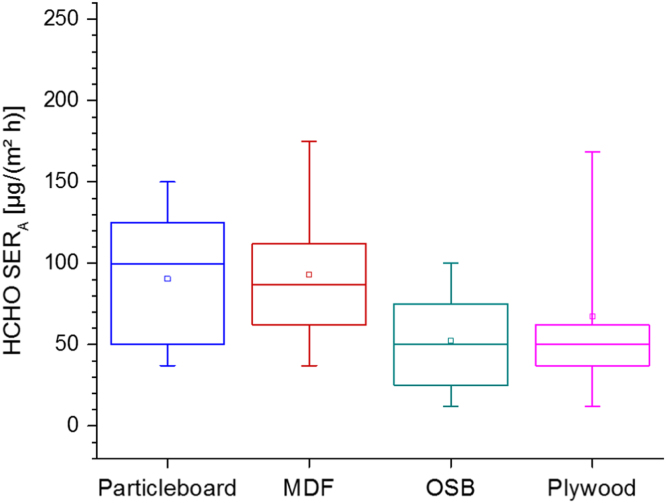
Box-Whisker plots (10−P, 25−P, 50−P (median), 75−P, 90−P and (□) mean) of calculated area specific emission rates of wood-based materials. Data from Table 28.

**Fig. 16:**
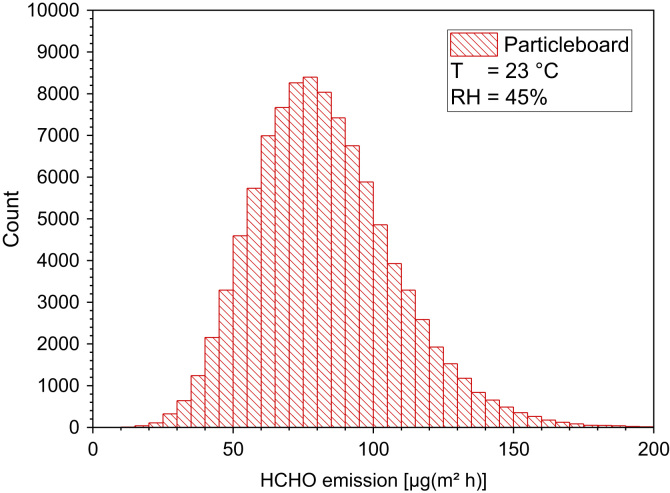
Monte-Carlo simulation of an asymmetric distribution of formaldehyde emission rates from particleboard with 100,000 runs and a statistical interval of Δ(HCHO) = 5 µg/(m² h). The statistical parameters are as follows: 25−P = 66 µg/(m² h), 50−P (median) = 83 µg/(m^2^ h), 75−P = 99 µg/(m² h), GM = 79 µg/(m² h) and *σ*_g_ = 1.37 µg/(m² h). ORIGIN LabTalk: exp[normal(100,000) *0.2+4.79]-[normal(100,000)*4+40].

**Fig. 17:**
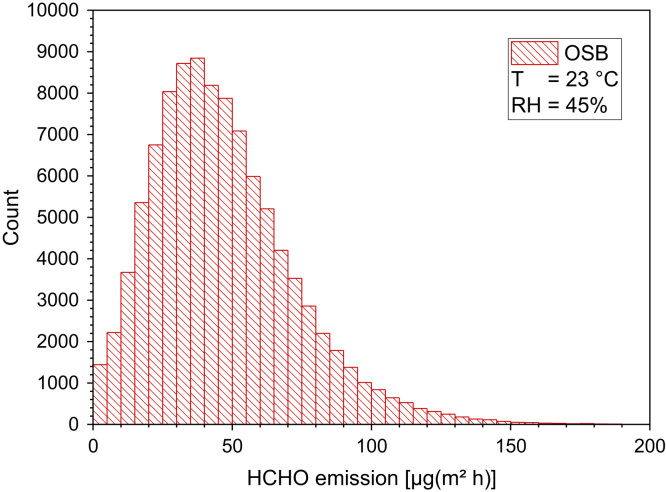
Monte-Carlo simulation of an asymmetric distribution of formaldehyde emission rates from OSB with 100,000 runs and a statistical interval of Δ(HCHO) = 5 µg/(m² h). The statistical parameters are as follows: 25−P = 29 µg/(m² h), 50−P (median) = 43 µg/(m^2^ h), 75−P = 61 µg/(m² h), GM = 39 µg/(m² h) and *σ*_g_ = 1.96 µg/(m² h). ORIGIN LabTalk: ABS(exp[normal(100,000) *0.32+4.29]–[normal(100,000)*4+30]).

Table 28 and Fig. 15, Fig. 16, Fig. 17.

**Table 28:** Formaldehyde area specific emission rates for different types of raw wood-based materials at 23 °C and 45% relative humidity. The data (in ppm) are taken from Marutzky and Schripp [87] and converted into µg/(m² h) under assumption of steady-state conditions.

**No.**	**SER**_**A**_**from particleboard [µg/(m² h)]**	**SER**_**A**_**from MDF [µg/(m² h)]**	**SER**_**A**_**from OSB [µg/(m² h)]**	**SER**_**A**_**from plywood [µg/(m² h)]**
1	50	100	100	12
2	125	87	75	12
3	137	25	75	12
4	112	112	75	62
5	112	87	87	62
6	125	150	62	12
7	37	37	87	62
8	37	50	25	50
9	125	62	37	37
10	125	175	12	50
11	150	100	12	37
12	37	75	50	50
13	50	50	25	37
14	50	25	50	50
15	112	112	50	50
16	125	100	62	50
17	200	25	100	37
18	125	125	112	50
19	125	75	62	12
20	62	212	100	25
21	25	50	100	50
22	150	62	37	25
23	50	225	12	150
24	12	175	50	187
25	12	125	50	125
26	137	87	37	62
27	112	62	12	237
28	112	75	125	150
29	125	62	6	62
30	37	75	12	200
31	37	100	75	
32	125		6	
33	125		100	
34	62		75	
35	50		75	
36	37		75	
37	37		87	
38	137		62	
39	75		87	
40	87			
41	62			
42	112			
43	50			
44	62			
45	175			
46	37			
47	62			
48	212

Yrieix et al. [88] published results of a European inter-laboratory comparison on raw particleboard. The mean of area specific emission rates from six independent laboratory results was 58.5 µg/(m² h) with a relative standard deviation of 9.6%. Horn et al. [80] measured the formaldehyde emission from seven OSB and found a range from 7 µg/(m² h) to 88 µg/(m² h) with a 50−P value of 33 µg/(m² h).

## 23 Formaldehyde from furniture

**Fig. 18:**
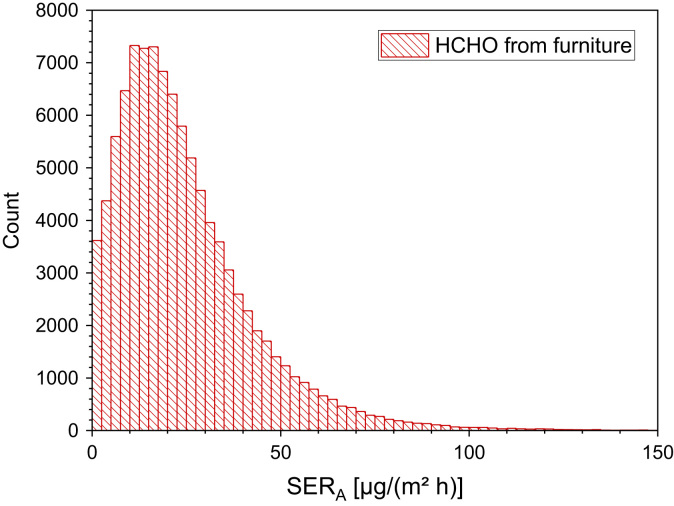
Monte-Carlo simulation of a log-normal + normal distribution of formaldehyde emission rates from furniture with 100,000 runs and a statistical interval of Δ(HCHO) = 2.5 µg/(m² h). The statistical parameters are as follows: 25−P = 11.7 µg/(m² h), 50−P (median) = 20.4 µg/(m^2^ h), 75−P = 32.7 µg/(m² h), GM = 17.8 µg/(m² h) and *σ*_g_ = 2.54 µg/(m² h). ORIGIN LabTalk: ABS(0.25*(exp[normal(100,000)*0.5+4.8]−[normal(100,000)*4+40])).

Table 29, Table 30 and Fig. 18.

**Table 29:** Test chamber conditions and unit specific emission rates in the study by Galinkina et al. [89].

**Object**	***T* [°C]**	**RH [%]**	**V [m³]**	**ACH [h**^**−1**^**]**	***L* [m²/m³]**	**SER**_**U**_**(168 h) [µg/h]**
Table plate	23	50	3	0.51	0.51	84
Office chair	23	50	3	0.67	1 Object	50

**Table 30:** Measured furniture, type of chamber, testing time and area specific emission rates in the study by Andersen et al. [90].

**ID**	**Type**	**Chamber volume [m³]**	**Testing time [d]**	**SER**_**A**_**[mg/m² h]**
1	Stool	0.225	28	0.18
2	Chair	0.225	28	0.10
3	Kitchen front door	0.225	6	<LOD
4	Kitchen front door	0.225	6	0.01
5	Kitchen front door	0.225	6	0.01
6	Kitchen front door	0.225	19	0.01
7	Kitchen front door	0.225	28	0.15
8	Kitchen front door	0.225	10	0.03
9	Coffee table	1	6	<LOD
10	Bookcase	1	6	0.02
11	Armchair	1	7	<LOD
12	Cabinet	15	5	0.02
13	Armchair	15	5	0.03
14	Chest of drawers	15	4	0.02
15	Armchair	15	5	<LOD
16	Dining table	15	5	<LOD
17	Bookcase	15	6	0.02
18	Bookcase	15	7	0.02
19	Armchair	24	5	0.05
20	Bookcase	24	6	0.01
21	Cabinet	24	6	0.02

## 24 Formaldehyde from laminate

**Fig. 19:**
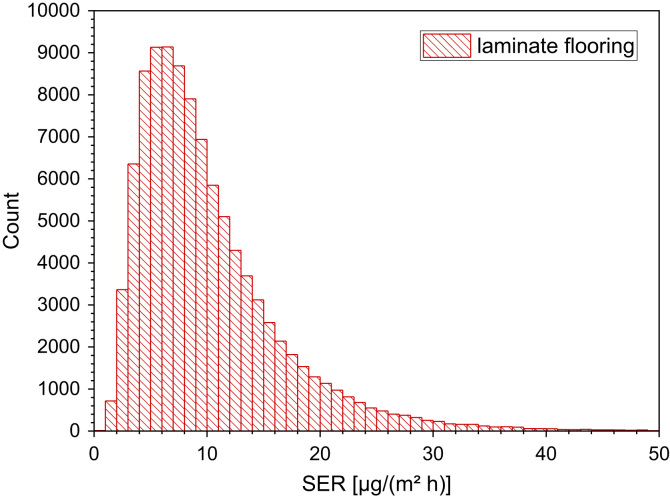
Monte-Carlo simulation of a log-normal distribution of formaldehyde emission rates from laminate flooring with 100,000 runs and a statistical interval of Δ(HCHO) = 1 µg/(m² h). The statistical parameters are as follows: 25−P = 5.7 µg/(m² h), 50−P (median) = 8.5 µg/(m^2^ h), 75−P = 12.7 µg/(m² h), GM = 8.5 µg/(m² h) and σ_g_ = 1.8 µg/(m² h). ORIGIN LabTalk: exp[normal(100,000)*0.6+2.14].

An et al. [92] studied the release of formaldehyde in a 20 l chamber and in the Field and Laboratory Emission Cell (FLEC) at *T* = 25 °C and RH = 50%, respectively. After 7 days testing time the calculated formaldehyde emission rates were between 7 µg/(m² h) and 15 µg/(m² h) (Table 31, Table 32 and Fig. 19).

**Table 31:** Chamber testing of laminate, formaldehyde steady-state concentrations after 28 days testing time (see Marutzky [91] for details).

**Carrier**	***T* (°C)**	**RH (%)**	**ACH (h**^**−1**^**)**	***L* (m²/m³)**	***C* (ppm)**
MDF	23	45	1	1	0.03
MDF	23	45	1	1	0.01
MDF	23	45	1	1	0.02
HDF	23	45	1	1	0.03
HDF	23	45	1	1	0.01
HDF	23	45	1	1	0.005
particleboard	23	45	1	1	0.03
particleboard	23	45	1	1	0.03

**Table 32:** Formaldehyde chamber testing of laminate flooring, steady-state concentrations (unpublished results, all measurements later than 2012).

**No.**	***T* [°C]**	**RH [%]**	**ACH [h**^**−1**^**]**	***L* [m²/m³]**	***t* [d]**	***C* [µg/m³]**	***C* [µg/(m² h)]**
1	23	50	0.5	0.4	3	8.8	7
2	23	50	0.5	0.4	3	<3.8	<3
3	23	50	0.5	0.4	7	<3.8	<3
4	23	50	0.5	0.4	28	5	4
5	23	50	0.5	0.4	3	3.8	3
6	23	50	0.5	0.4	3	5	4
7	23	50	0.5	0.4	3	8.8	7
8	23	50	0.5	0.4	7	8.8	7
9	23	50	0.5	0.4	3	16.3	13
10	23	50	0.5	0.4	7	16.3	13
11	23	50	0.5	0.4	28	12.5	10
12	23	50	0.5	0.4	3	6.3	5
13	23	50	0.5	0.4	7	6.3	5
14	23	50	0.5	0.4	28	6.3	5
15	23	50	0.5	0.4	3	11.3	9
16	23	50	0.5	0.4	7	11.3	9
17	23	50	0.5	0.4	28	12.5	10
18	23	50	0.5	0.4	3	30	24
19	23	50	0.5	0.4	7	30	24
20	23	50	0.5	0.4	28	28.3	23
21	23	50	0.5	0.4	3	35	28
22	23	50	0.5	0.4	7	32.5	26
23	23	50	0.5	0.4	3	10	8
24	23	50	0.5	0.4	3	11.3	9
25	23	50	0.5	0.4	3	5	4
26	23	50	0.5	0.4	7	5	4
27	23	50	0.5	0.4	28	3.8	3

Pierce et al. [93] investigated the impact of laminate flooring manufactured in China on formaldehyde concentrations in a model room. In complementary chamber tests with two selected products and under so-called non-destructive test conditions the chamber concentrations after seven days were 0.018 ppm (product 1) and 0.012 ppm (product 2). The test conditions were *T* = 25 °C, (77 °F), RH = 50%, ACH = 0.5 h^−1^, *L*= 0.43 m²/m³.

The Centers for Disease Control and Prevention (CDC) [94] released a report on formaldehyde emission from Chinese−produced laminate. Increased emission rates with a geometric mean of 41.7 µg/(m² h), a geometric standard deviation of 2.3 µg/(m² h) and maximum value of 350 µg/(m² h) at *T* = 24.5 – 25.7 °C and RH = 46.0 – 51.5% are reported.

Wiglusz et al. [95] studied the effect of temperature on the emission of formaldehyde from laminate flooring. The tested materials did not show formaldehyde emissions at temperatures of 23 °C and 29 °C. At 50 °C one of the materials showed a formaldehyde emission rate of approx. 40 µg/(m² h) after 20 days testing time.

## 25 Formaldehyde from windows and doors

Table 33, Table 34.

**Table 33:** Types of measured windows, chamber conditions and area specific emission rates. The data were taken from the study by Wensing and Bliemetsrieder [96].

**Material**	***T* [°C]**	**RH [%]**	**ACH [h**^**−1**^**]**	***L* [m²/m³]**	**HCHO [µg/m³]**
Spruce (coated)	23	50	0.57	0.076	<2
Larch (coated)	23	50	0.57	0.076	<2
Spruce (coated)	23	50	0.57	0.076	<2
Spruce (coated)	23	50	0.57	0.076	<2
Spruce (coated)	23	50	0.57	0.076	<3
Spruce (coated)	23	50	0.46	0.061	<3
Spruce (coated)	23	50	0.46	0.061	<3

**Table 34:** Types of measured door leafs and door frames, chamber conditions and area specific emission rates. The data were taken from the study by Wensing and Bliemetsrieder [97]. See also Wensing et al. [98].

**Material**	***T* [°C]**	**RH [%]**	**ACH [h**^**−1**^**]**	***L* [m²/m³]**	**SER**_**A**_**[µg/(m² h)]**	**Time [d]**
Door leaf	23	50	0.5	1.09	50.9	28
Door leaf	23	50	0.5	1.09	30.7	28
Door leaf	23	50	0.5	1.09	41.7	28
Door leaf	23	50	0.5	1.09	39.4	28
Door leaf	23	50	0.5	1.09	9.6	28
Door leaf	23	50	0.5	1.09	2.3	28
Door leaf	23	50	0.5	1.09	7.3	28
Door leaf	23	50	0.5	1.09	23.9	28
Door leaf	23	50	0.5	1.09	22.5	14
Door leaf	23	50	0.5	1.09	9.2	28
Door leaf	23	50	0.5	1.09	21.6	28
Door leaf	23	50	0.5	1.09	4.1	28
Door leaf	23	50	0.5	1.09	20.2	28
Door leaf	23	50	0.5	1.09	13.3	14
Door leaf	23	50	0.5	1.09	65.6	28
Door leaf	23	50	0.5	1.09	38.1	28
Door leaf	23	50	0.5	1.09	10.6	28
Door leaf	23	50	0.5	1.09	5.0	28
Door leaf	23	50	0.5	1.09	4.1	28
Door leaf	23	50	0.5	1.09	53.7	28
Door leaf	23	50	0.5	1.09	32.1	28
Door leaf	23	50	0.5	1.09	3.7	28
Door leaf	23	50	0.5	1.09	53.2	28
Door leaf	23	50	0.5	1.09	51.4	28
Door leaf	23	50	0.5	1.09	25.7	14
Door leaf	23	50	0.5	1.09	51.4	28
Door frame	23	50	0.5	1.09	120.2	28
Door frame	23	50	0.5	1.09	211.9	28
Door frame	23	50	0.5	1.09	100.9	28
Door frame	23	50	0.5	1.09	284.4	28
Door frame	23	50	0.5	1.09	98.2	28
Door frame	23	50	0.5	1.09	76.1	28
Door frame	23	50	0.5	1.09	2.8	28

## 26 Formaldehyde from mineral wool

**Fig. 20:**
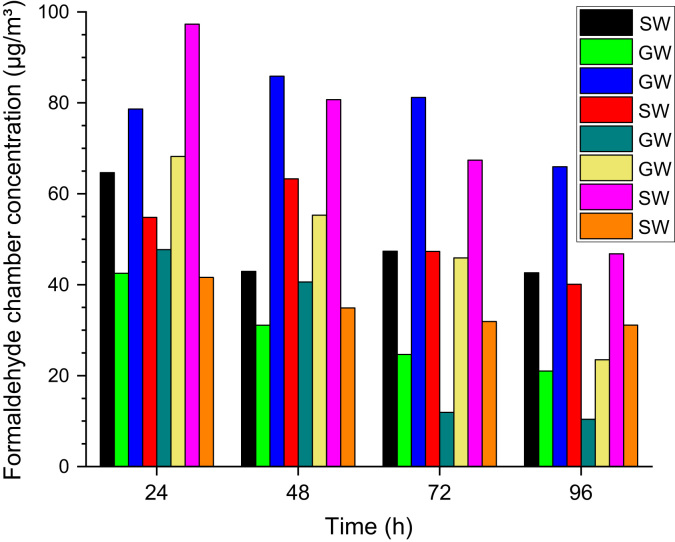
Formaldehyde concentrations in the 1 m³ test chamber after 24–96 h testing time. Four samples were made of stone wool (SW) and four samples were made of glass wool (GW). The chamber conditions were *T* = 23 °C, RH = 50%, ACH = 1 h^−1^ and *L* = 1 m²/m³.

An inter laboratory comparison experiment on the determination of formaldehyde emitted from mineral wool board using small test chambers has been described by Wiglusz et al. [99]. Eleven laboratories took part and the most reliable testing round yielded a range between 44 µg/(m² h) and 210 µg/(m² h) with a 50−P value of 57 µg/(m² h). So far unpublished WKI data from eight different samples of mineral wool (four glass wool, four stone wool) are shown in Fig. 20. The concentrations after 96 h were between 10 µg/m³ and 66 µg/m³ with a geometric mean of 31.0 µg/m³.

## 27 Aging effect

**Fig. 21:**
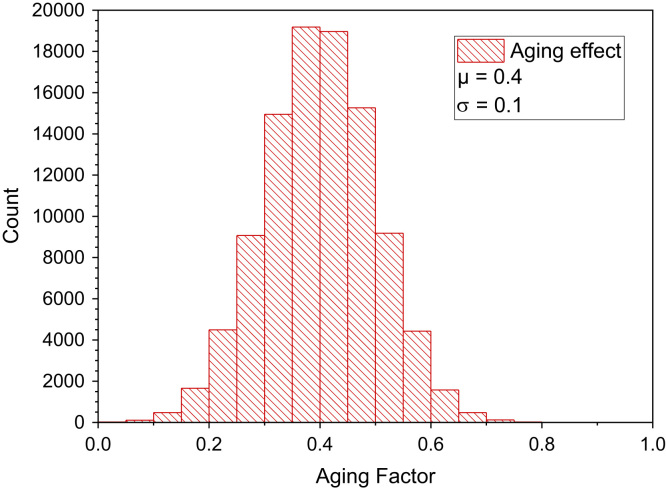
Monte-Carlo simulation of a normal distribution of an aging factor with 100,000 runs and a statistical interval of Δ = 0.05. The statistical parameters are *µ* = 0.4 and *σ* = 0.1. ORIGIN LabTalk: normal(100,000)*0.1+0.4.

Few studies deal with the long-term emission behavior of materials and products. Most available data refer to test chamber results of freshly produced materials measured after 28 days. Colombo et al. [100] applied an empirical potential function to extrapolate the formaldehyde emission rate of particleboard, fiberboard and plywood in large environmental chambers. For plywood, taking the 28 days value as a starting point, reductions of 33% after 1 year and 42% after 2 years can be calculated from the fit parameters. For particleboard, taking again the 28 days value as a starting point, reductions of 45% after 1 year and 66% after 2 years are obtained from the fit parameters. Brown [101] studied the release of formaldehyde from particleboard and MDF in test chambers and found that formaldehyde emission factors for all products assessed were approximately 300–400 mg/(m² h) in the first few weeks after product manufacture and 80–240 mg/(m² h) after 6–10 months. Liang et al. [102] studied the long-term formaldehyde emissions from MDF in a full-scale experimental room and found that concentrations decreased by 20–65% in the corresponding months of the second year. Under the assumption that the lifetime of wood-based materials in housing is ten years or more, a weighting factor of 0.4 can be estimated. Fig. 21 shows a Monte-Carlo simulation under assumption of a normal distribution.

## 28 Source/sink behavior and diffusion effects

**Fig. 22:**
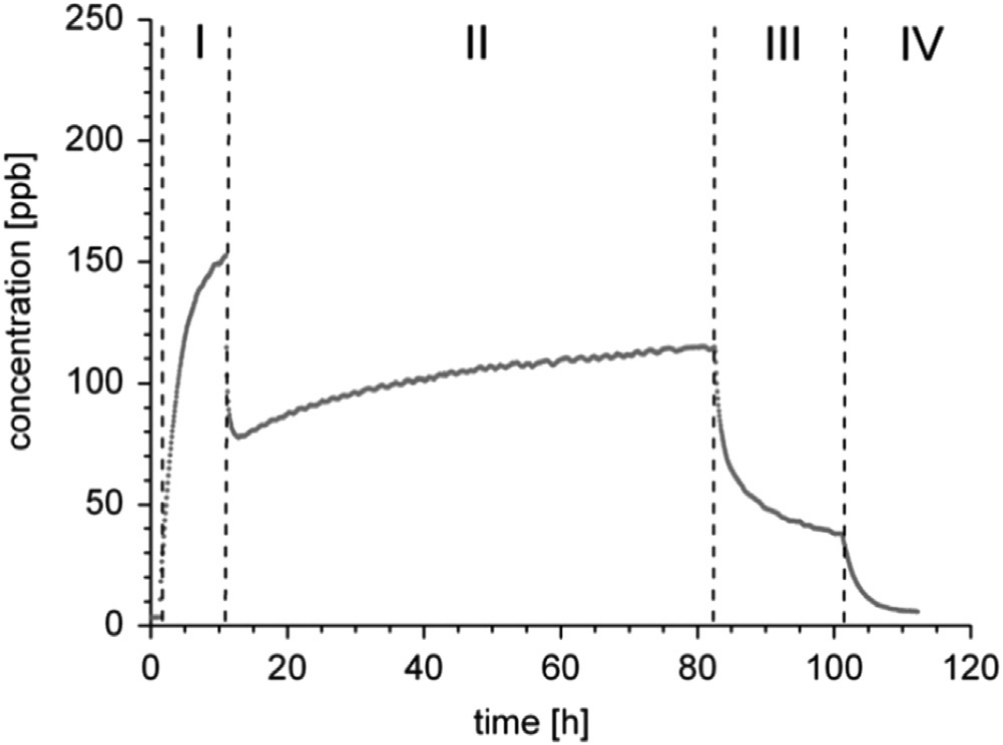
Formaldehyde sorption/desorption experiment with a ceiling tile (mineral wool covered with glass fleece and paint) in a 1 m³ glass chamber. The figure was taken from Gunschera et al. [103]. Phase I: formaldehyde was doped from a gas bottle into an empty 1 m³ glass chamber for 4–6 h to achieve a steady-state concentration of approx. 150–160 ppb. Phase II: the chamber was loaded with the test specimen, loading factor 0.5 m²/m³, *T* = 23 °C, RH = 50%, ACH = 0.4 h^−1^ and the formaldehyde concentration was continuously monitored for 70–75 h. Phase III: the formaldehyde supply was stopped and formaldehyde monitoring was continued for 24 h. Phase IV: the chamber was emptied and the decay of the formaldehyde concentration was measured for 12 h.

**Fig. 23:**
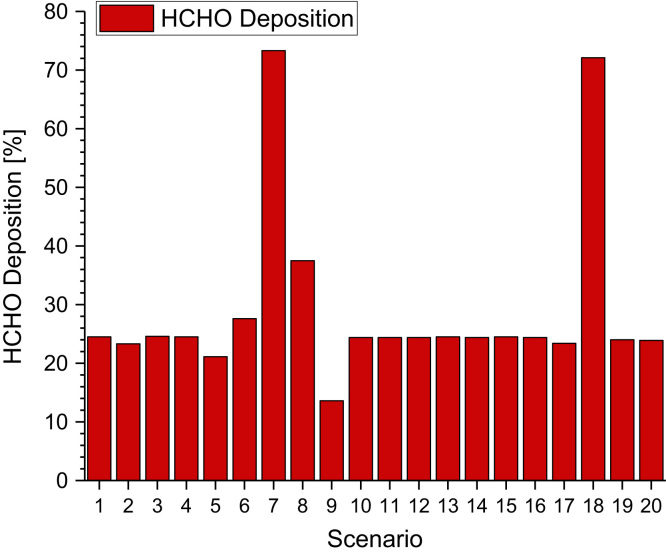
Percentage of deposition of formaldehyde for 20 different indoor scenarios (the data are taken from Mendez et al. [36]).

**Fig. 24:**
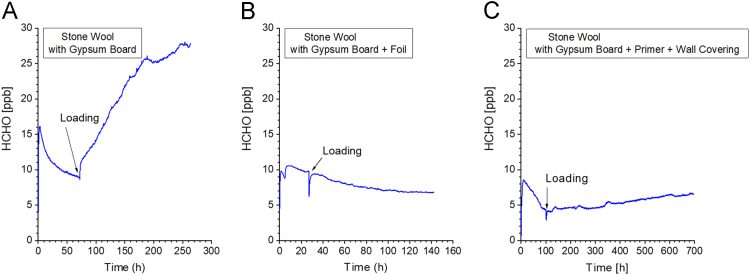
Formaldehyde concentration plots in three different diffusion experiments. The figure was taken from Gunschera et al. [103]. A tray made from stainless steel was completely filled with commercially available glass wool or stone wool several kinds of mineral wool and capped with a pre-conditioned 0.01 m gypsum board. The gap between tray and board was sealed and fixed in a metal frame. This construction was set up in a 1 m³ glass chamber at *T* = 23 °C, RH = 50% and ACH = 0.5 h^−1^. If the mineral wool is only covered with gypsum board (A), a diffusion of formaldehyde into the chamber air is clearly visible, leading to a steady-state concentration of about 30 ppb. If the surface of the gypsum board is covered with foil (B) the decaying concentration curve proves that the diffusion effect is negligible. In case of gypsum board being treated with primer and wallcovering (C) a very slight increase of the formaldehyde concentration could be observed (3 ppb within 600 h testing time).

Many other studies have shown that materials like textiles, wool, zeolites, etc. act as strong but partly reversible sources for formaldehyde [104], [105], [106], [107] (Fig. 22, Fig. 23, Fig. 24).

The barrier effect was also investigated by Yrieix [110], [111], [112] for different types of wood-based materials. One study [112] focused on formaldehyde emissions from different coated particleboards (melamine faced board with two paper basis weights, laminate board, wood veneer with two porosities, not varnished finish foil). In a follow-up study Yrieix compared the barrier effect of melamine impregnated decorative papers to formaldehyde emissions according to their paper basis weight (low and high basis weight) and to paper printing (surface printing or in the mass of the paper, mineral content) [110] (Table 35, Table 36, Table 37).

**Table 35:** Formaldehyde concentrations in the test chamber under the conditions of EN 16516 [108] (*T* = 23 °C, RH = 50%, ACH = 0.5 h^−1^, *L* = 1 m²/m³). See Meyer et al. [109] for details.

**Construction**	***C* [ppm]**
Particleboard	0.13
Particleboard + gypsum plasterboard	0.10
Particleboard + gypsum plasterboard + woodchip wallpaper + paint	0.08
Particleboard + gypsum plasterboard + vinyl wallpaper	0.05
Particleboard + diffusion barrier film + gypsum plasterboard + vinyl wallpaper	0.02

**Table 36:** Reduction of the area specific formaldehyde emission rate from particleboard by different types of covering (1 m³ stainless steel chamber, *T* = 23 °C, RH = 45%, ACH = 0.5 h^−1^ and *L* = 0.5 m²/m³). WKI, unpublished data.

**Test**	**Covering**	**Rel. SER**_**A**_**[%]**	**Reduction of rel. SER**_**A**_**[%]**
1	No covering	100	0
2	With primer	30	70
3	With primer and dispersion paint	24	76
4	With primer and plaster	22	78
5	With primer and wallpaper (fleece)	6	94
6	With primer and latex paint	2	98

**Table 37:** Formaldehyde emission rates of raw wood-based materials and covered wood based materials after 28 d. The effect of formaldehye reduction is also presented. The data are taken from Yrieix [110].

**Test no.**	**Raw material [µg/(m² h)]**	**Covered material [µg/(m² h)]**	**Reduction [%]**
01	82	3.3	96
02	82	3.5	96
03	107	15	86
04	42	3.0	93
05	107	23	79
06	42	8.0	81
07	60	2.5	96
08	75	4.0	95
09	50	5.0	90
10	20	4.1	80
11	34	4.7	86
12	115	12	90
