# Supplementary material for: Data on formaldehyde sources, formaldehyde concentrations and air exchange rates in European housings
Source: Data Brief. 2018 Nov 24;22:400–35. doi: 10.1016/j.dib.2018.11.096 (PMC6309026; doi:10.1016/j.dib.2018.11.096)
Supplement: Supplementary file 1 — Supplementary material [file mmc1.pdf]

CONFLICT OF INTEREST STATEMENT

Data-in-Brief

Manuscript No.: DIB-D-18-02605

Title: Data on formaldehyde sources, formaldehyde concentrations and air exchange rates in European housings Journal Title: Data in Brief Corresponding Author: Professor Tunga Salthammer All Authors: Tunga Salthammer Submit Date: Sep 27, 2018

The author certifies that he has NO affiliations with or involvement in any organization or entity with any financial interest (such as honoraria; educational grants; participation in speakers' bureaus; membership, employment, consultancies, stock ownership, or other equity interest; and expert testimony or patent-licensing arrangements), or non-financial interest (such as personal or professional relationships, affiliations, knowledge or beliefs) in the subject matter or materials discussed in this manuscript.

Braunschweig, 15. November 2018

Prof. Dr. Tunga Salthammer
